# Driving With Agents: Investigating the Influences of Anthropomorphism Level and Physicality of Agents on Drivers' Perceived Control, Trust, and Driving Performance

**DOI:** 10.3389/fpsyg.2022.883417

**Published:** 2022-06-15

**Authors:** Peiyao Cheng, Fangang Meng, Jie Yao, Yiran Wang

**Affiliations:** ^1^School of Humanities and Social Sciences, Harbin Institute of Technology (Shenzhen), Shenzhen, China; ^2^Efficiency Application Department, Kuaishou Technology, Beijing, China

**Keywords:** agents, anthropomorphism, driving, physicality, perceived control, trust

## Abstract

As technological development is driven by artificial intelligence, many automotive manufacturers have integrated intelligent agents into in-vehicle information systems (IVIS) to create more meaningful interactions. One of the most important decisions in developing agents is how to embody them, because the different ways of embodying agents will significantly affect user perception and performance. This study addressed the issue by investigating the influences of agent embodiments on users in driving contexts. Through a factorial experiment (*N* = 116), the effects of anthropomorphism level (low vs. high) and physicality (virtual vs. physical presence) on users' trust, perceived control, and driving performance were examined. Results revealed an interaction effect between anthropomorphism level and physicality on both users' perceived control and cognitive trust. Specifically, when encountering high-level anthropomorphized agents, consumers reported lower ratings of trust toward the physically present agent than toward the virtually present one, and this interaction effect was mediated by perceived control. Although no main effects of anthropomorphism level or physicality were found, additional analyses showed that anthropomorphism level significantly improved users' cognitive trust for those unfamiliar with IVIS. No significant differences were found in terms of driving performances. These results indicate the influences of in-vehicle agents' embodiments on drivers' experience.

## Introduction

Driven by developments in artificial intelligence, an increasing number of intelligent agents have been developed and integrated into various contexts in everyday life. For instance, the agent “Alexa” has been integrated into smart speakers to assist users with domestic tasks. Intelligent agents exhibit a certain degree of autonomy and they can perceive and communicate with their surroundings (Ferber and Weiss, 1999). Automobile manufacturers also echo this trend by developing in-vehicle agents, which are also referred to as Driving Support Agent (DSA) (Tanaka et al., 2018a,b; Karatas et al., 2019; Lee et al., 2019; Miyamoto et al., 2021). Drivers can interact with in-vehicle agents [often integrated into in-vehicle information systems (IVIS)], through voice commandsin order to learn about driving-related information (e.g., traffic, weather conditions) and completing some secondary tasks (e.g., turning the air conditioner on/off, opening/closing windows). Some in-vehicle agents can even provide assistance for safer driving (e.g., lane keeping, speed control, fuel management). Through sensing, listening, and taking active roles to give advice, the in-vehicle agents can potentially transform the traditional human-machine interaction into human-human interaction, facilitating a natural and intuitive interaction.

Increasing research attention has been paid to developing intelligent agents in driving contexts for enriching the driving experience. Recent studies have explored the desirable personality of in-vehicle agents (Braun et al., 2019), the utterance of in-vehicle agents influences drivers' acceptance in self-driving (Miyamoto et al., 2021) and autonomous driving contexts (Lee et al., 2019). However, besides in-vehicle agents' conversational styles, agent embodiments as another important factor, also deserve more research attention. Agent appearance has been found to significantly influence user experience (Shiban et al., 2015; Abubshait and Wiese, 2017; Ter Stal et al., 2020a). Agents can be created to resemble humans, animals, objects, robots, or mystical creatures (Straßmann and Krämer, 2017). They can also be virtual or physical, i.e., created as a virtual character that is only presented on digital screens, or physically with tangible materials and structure (Li, 2015).

In fact, agents are supported by sophisticated algorithms. Designers and developers have the freedom to embody agents in various ways and thereby deliberately influence user experience. Different agent appearances can trigger users to interact with agents differently. Therefore, it is crucial to understand how different agent embodiments influence user responses in driving contexts. This study specifically focused on two dimensions of agent embodiments (Ziemke, 2001): anthropomorphism level (low vs. high) and physicality (virtual vs. physical).

Prior studies have demonstrated the significant influences of agents' anthropomorphism and physicality on user experience and performance. For instance, anthropomorphised agents were found to improve users' perceived enjoyments and trust of online shopping websites (Luo et al., 2006; Qiu and Benbasat, 2009), and student performance in online learning contexts (Li et al., 2016). Agent physicality could also improve user trust (Wainer et al., 2006; Kiesler et al., 2008), enjoyments (Wainer et al., 2007; Kose-Bagci et al., 2009), and attitudes (Kiesler et al., 2008). However, few studies have investigated the interaction effects between anthropomorphism level and physicality, while examining the interactions among different layers of agent embodiments has drawn more and more research attention, such as the study on the interaction between agency and anthropomorphism (Nowak and Biocca, 2004; Kang and Kim, 2020), the interplay of bodily appearance and movement (Castro-González et al., 2016), and the interaction between anthropomorphism and realism (Li et al., 2016). This study aimed to extend this line of research by investigating the interaction effects between anthropomorphism level and physicality in driving contexts.

Furthermore, past research has pointed out that the influences of agent embodiments are specific to task and interaction characteristics (van Vugt et al., 2007; Hofmann et al., 2015; Schrader, 2019). Schrader (2019) found that people created virtual agents differently for learning and entertainment contexts. Thus, it is still questionable whether these findings on agents' anthropomorphism and physicality found in other contexts (e.g., online learning, online shopping, smart home contexts) can be applicable in driving contexts. Considering the differences between driving and other contexts, it is necessary to examine how agents' anthropomorphism level and physicality influence driving experience and performance, which can contribute to current literature by considering the influence of specific task contexts.

**Figure 1:**
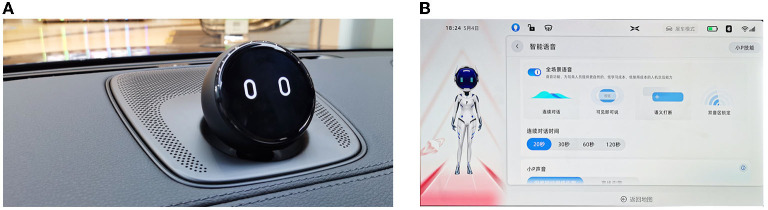
Examples of agents in different forms: **(A)** NOMI in the physical form; **(B)** XiaoP in the virtual form.

From a practical perspective, this topic is also worthy of investigation given the two important factors for developers to decide while embodying agents. Different ways of agent embodiments exist in the current practices of automobile manufacturers. For example, the Chinese manufacturer NIO developed the in-vehicle agent NOMI, which is physically located in the vehicle with a shape resembling the human head and can exhibit facial expressions (see Figure 1A). Another Chinese manufacturer XiaoPeng Motors developed an agent named XiaoP, which is virtually presented on the screen, and highly resembles a person with multiple human-like characteristics, such as facial expressions, head, body, arms, and legs (see Figure 1B). Therefore, it is crucial to understand how agents' anthropomorphism and physicality will indeed influence user experience.

## Related Works

### Users' Perception of IVIS and Driving Performance

In-vehicle information systems is indispensable for users' driving, which becomes increasingly complex nowadays because of the integration of newly-added functions (e.g., advice-giving function, voice interaction function). Complex and sophisticated IVIS can cause user resistance for effective usage (Kim and Lee, 2016), and therefore user trust of IVIS becomes crucial. In fact, prior research has shown that user trust is a precondition for their effective usage of complex and sophisticated systems (Lee and See, 2004). Trust largely alleviates users' anxiety triggered by the increasingly complicated systems. Users are more likely to use systems that they trust while avoiding systems that they do not trust. Greater trust has been demonstrated to improve users' usage of voice interaction technology (Nguyen et al., 2019), mobile payment system (Yan and Yang, 2015), and autonomous vehicle (Choi and Ji, 2015). Furthermore, trust is a multi-dimension concept (Soh et al., 2009), which involves the users' belief about the credibility of information given by a system and users' subjective feelings toward the system. To capture the different facets, cognitive and affective dimension are proposed. Cognitive trust relates rational processing and focuses on the users' evaluation of credibility of given information, while affective trust refers to the users' subjective feeling toward a system (Lewis and Weigert, 1985; Soh et al., 2009).

Perceived control is an important factor that influences user trust toward a system. Perceived control refers to one's subjective evaluation of his or her ability to exert influence over the environment or systems (Bowen and Johnston, 1999). It relates to a situational perceived ability to affect the outcome of a system (Pacherie, 2007; Pacheco et al., 2013). People favor situations that they can control while avoiding situations they cannot control (Klimmt et al., 2007). The positive influences of perceived control have been demonstrated in the context of online games (Wang, 2014) and mobile health service systems (Zhao et al., 2018). In driving contexts, perceived control closely relates to driving experience and driving performance (Murata et al., 2016; Wen et al., 2019). Users' perceived control is influenced by the functions provided by IVIS. Certain driving support functions (e.g., lane-keeping monitor) decreases the drivers' sense of agency (Yun et al., 2018), which hinders drivers' sense of control, leading to drivers' disengagement (Navarro et al., 2016) and threatens driving safety (Oviedo-Trespalacios et al., 2019). The involvement of in-vehicle agents can also potentially influence the users' perceived control, which further affects user's trust of IVIS. Depending on different agent embodiments (i.e., anthropomorphism level and physicality), users may respond to IVIS differently and be affected in user experience and performance.

### The Influences of Agents' Anthropomorphism

Anthropomorphism refers to the attribution of humanlike characteristics (e.g., human forms, voices, behaviors) to inanimate, artificial agents such as robots and agents (Bartneck et al., 2009; Waytz et al., 2014). While interacting with computers and systems, people mindlessly apply interpersonal social rules as if they interact with human beings (Nass et al., 1995; Reeves and Nass, 1996). The involvement of anthropomorphism reinforces the tendency to interact with computers and systems in social ways, which further bring natural interactions in various contexts. For instance, anthropomorphized agents have improved users' engagement and attitudes toward learning systems (Moundridou and Virvou, 2002; Chang et al., 2010), users' enjoyment and trust of e-commerce websites (Qiu and Benbasat, 2009), user trust in diabetes decision-support aid systems (Pak et al., 2012), and users' trust of autonomous vehicles (Lee et al., 2015).

With the acknowledgment of benefits of the anthropomorphism strategy, a large number of studies have been conducted to further explore the optimal level of anthropomorphism. Should an agent be highly similar to a human being? Or should limited anthropomorphic cues (e.g., head, facial expression, body) be sufficient? Thus far, the findings are inconsistent. On the one hand, prior research concluded that as users generally consider agents as independent social actors, the similarities between an agent and a user are positively correlated with user responses (Sundar and Nass, 2000). Following this, the increasing similarities will trigger users to respond to the agents more socially. For instance, prior research found that, in comparison to low-level anthropomorphized agents, high-level anthropomorphized agents improved consumer trust and attitudes to e-commerce websites (Luo et al., 2006). Gong (2008) further compared agents of four varying anthropomorphism levels progressing from low-, medium-, high-anthropomorphism to real human pictures, and found that users' trust improved along with the increase of anthropomorphism level.

On the other hand, the uncanny valley theory pointed out that high-level anthropomorphism can be detrimental. Specifically, users respond to robots more positively with the increasing similarities between robots and a real person. However, users start to have negative responses when the robots become highly realistic (Mori et al., 2012). It is not easy to create super realistic anthropomorphized agents to reach the reverse point. Nevertheless, there is evidence showing that anthropomorphized agents make negative influences in certain contexts. In game playing contexts, the presence of an anthropomorphized computer helper threatens users' perceived control. Consequently, users experienced less enjoyment from playing the games (Kim et al., 2016). Prior research further supported that users' adoption of task-specific advice has not been influenced by agents that exhibited different anthropomorphism levels (i.e., no, low-, high-level anthropomorphism agent), although they reported higher trust levels toward high-level anthropomorphism agents (Kulms and Kopp, 2019).

Furthermore, the influences of an agent's anthropomorphism level are likely to be moderated by users' individual differences. In fact, prior research has stated that a users' own characteristics can moderate the influences of anthropomorphism level (Złotowski et al., 2015). People's tendency to anthropomorphize agents increases with their motivation to understand the agents (Epley et al., 2007). When users lack understanding of a system, it is more likely for users to anthropomorphize agents. When people have a comprehensive understanding of systems, the tendency of anthropomorphizing agents can be largely reduced.

### The Influence of Agents' Physicality

Agents can be presented to users physically or virtually, to which people will respond differently. In general, a physically present agent can provide better affordance, which is likely to trigger more social interactions with agents (Fong et al., 2003). In comparison to virtual presence, physical presence allows users to feel agents with richer senses, including vision, touch, and smell. As a result, users are more likely to consider the existence of a system as a real person and interact with a system as a real person (Lee et al., 2006, p. 10; Mann et al., 2015, p. 35).

Previous studies further found that physicality can improve user trust (Wainer et al., 2006; Kiesler et al., 2008), enjoyments (Wainer et al., 2007; Kose-Bagci et al., 2009), and attitudes (Kiesler et al., 2008). In driving contexts, similar findings were also reported. When encountering physical agents, users reported a stronger tendency of considering the agents as real persons (Lee et al., 2019) and form higher trust levels (Kraus et al., 2016). In terms of the influence of physicality on user performance, Li (2015) suggested that the presence of a physical robot will draw more user attention, which could lead to lower driving performance. However, in a mathematical puzzle-solving task, no significant influences of physicality were found in terms of users' performance (Hoffmann and Krämer, 2013).

### Research Questions

To summarize, previous studies have shown the respective influences of agent anthropomorphism level and physicality on user trust of agents. However, how agents' anthropomorphism level and physicality influence user trust synergistically remain unclear. Moreover, most of existing research on agents' anthropomorphism level and physicality was conducted in the contexts of healthcare (Mann et al., 2015) and online shopping (Luo et al., 2006). As interaction task characteristics largely influence user experience (van Vugt et al., 2007; Hofmann et al., 2015; Schrader, 2019), it is still questionable whether the findings on anthropomorphism level and physicality can be directly applicable to driving contexts. The current study addressed these questions by manipulating agents' anthropomorphism level and physicality on user trust and performance in driving contexts. Because of the inconsistent findings in current literature and the uniqueness of driving contexts, we did not give directional hypotheses but propose the following two research questions:

**RQ1**. How does agents' anthropomorphism level influence users' trust and driving performance?

**RQ2**. How does agents' physicality influence users' trust and driving performance?

Moreover, there is a possible interaction effect between anthropomorphism level and physicality. Specifically, as demonstrated in prior research, agents' anthropomorphism level increases similarities between agents and humans, which trigger users to consider agents as real social actors (Gong, 2008). In comparison to virtual presence, physical presence further enhances the tendency (Lee et al., 2006), which may deliver a stronger feeling of “another person” rather than a “system.” As a result, the combination of high-level anthropomorphism and physicality can reinforce users' perception of “another person.” The strong sense of “another person” can bring benefits in the contexts when users seek help from systems, such as in online shopping contexts (Qiu and Benbasat, 2009) and decision-making assisted systems (Pak et al., 2012). However, users' strong sense of “another person” is not always beneficial. For instance, Bartneck et al. (2010) report that a stronger feeling of “another person” made users feel embarrassed in a medical examination, where users were asked to take off clothes for examination. The stronger feeling of “another person” reduces perceived enjoyments that people gained from game playing (Kim et al., 2016) and hinders users' performance in searching tasks (Rickenberg and Reeves, 2000).

However, in driving contexts, where users intend to have full control over the driving process (Murata et al., 2016; Wen et al., 2019), the stronger feeling of “another person being” may be detrimental because it may threaten users' perceived control. In other words, perceived control may serve as a mediator. Therefore, to understand how anthropomorphism level and physicality interact with each other as well as the role of perceived control, the following research question is proposed.

**RQ3**. How do agents' anthropomorphism level and physicality interactively influence users' perceived control, trust, and driving performance?

While investigating the influences of anthropomorphism level and physicality, we also consider the moderating role of users' familiarity with IVIS. As users' own characteristics can moderate the influences of anthropomorphism level (Złotowski et al., 2015), the influence of anthropomorphism level is likely to be significant for people who are unfamiliar with IVIS. Similarly, when users have limited understanding of a system, a physically present agent might be more desirable because of the stronger sense of “another person being.” Therefore, we proposed the following research question.

**RQ4**. How do users' familiarity with IVIS moderate the effects of agents' anthropomorphism level and physicality?

## Methods

### Design and Participants

To address the above research questions, a 2 × 2 factorial between-subjects experiment was designed and conducted, with agent anthropomorphism level (low vs. high) and agent physicality (virtual vs. physical) as independent variables. Participants were randomly assigned to one of the four conditions and were asked to complete the driving tasks and interact with agents during driving. The driving task and interaction tasks were identical among four conditions.

Participants were recruited for the experiment through volunteering to respond to online advertisements posted on a public university campus. One hundred sixteen participants were collected (62.93% male, mean age = 22.88). All the participants had qualified driving licenses and normal or corrected visual acuity. They received a small amount of compensation for their participation in the experiment.

### Apparatus and Stimuli

#### Apparatus

**Figure 2:**
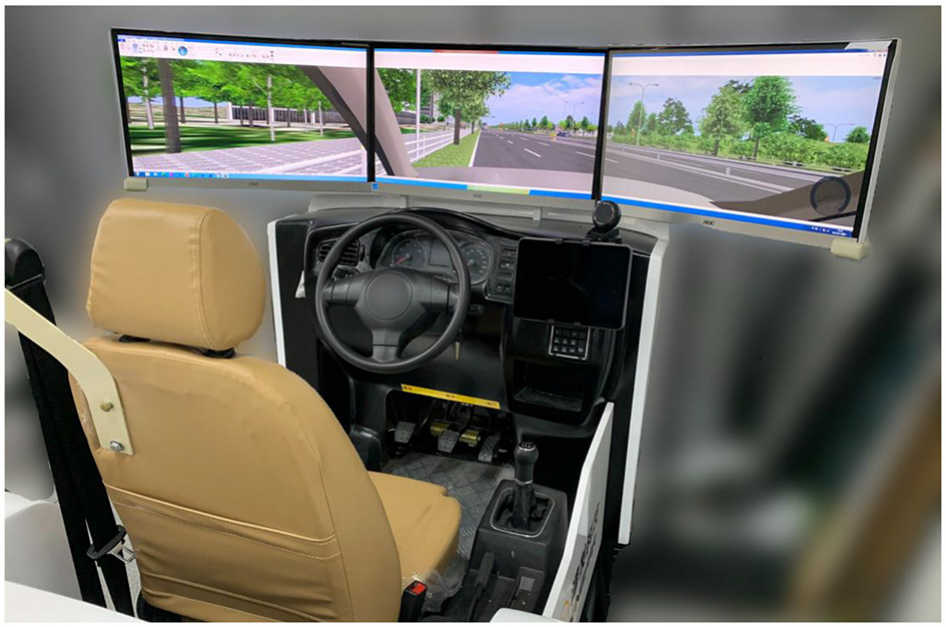
The driving simulator used in the experiment: the condition of a physical present agent with low-level anthropomorphism.

The experiment was conducted using a medium fidelity driving simulator (see Figure 2). The simulator provides a 135° field of view of the driving environment with three 27-inch monitors. In addition, to stimulate the visual interfaces of IVIS, a tablet was used with 10.2 inches screen and a resolution of 2160^*^1620. This tablet was positioned on the right side of the steering wheel. Participants were asked to drive along a route, which was created to resemble the typical roads in China. The driving route was around 4 km long, and consisted of a variety of scenarios, including stop-sign intersections (with/without crossing traffic), signalized intersections, car-following, and pedestrian crossing to resemble a real driving experience as much as possible. Participants drove in the right lane of the road.

#### Stimuli Creation

**Figure 3:**
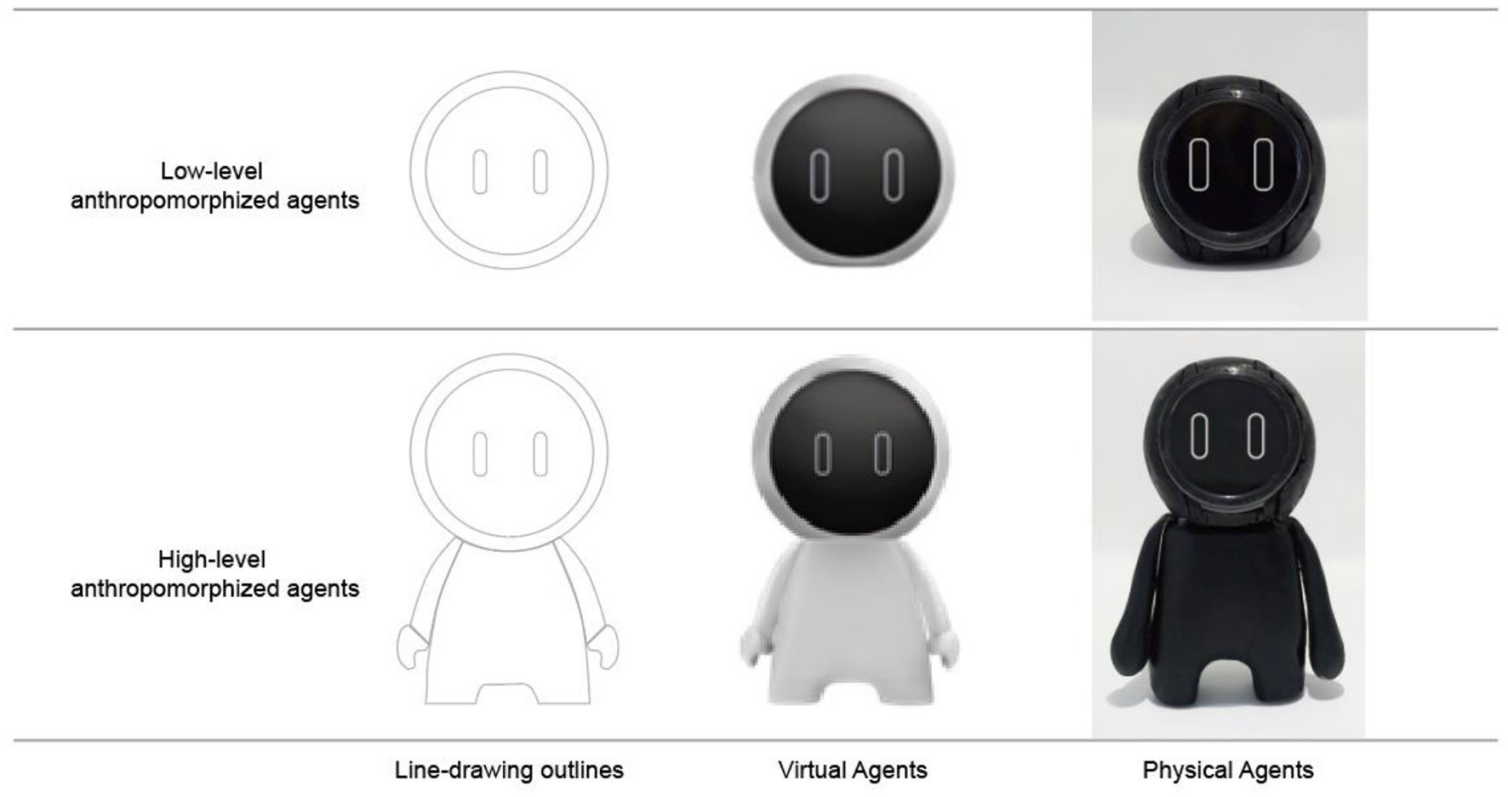
Stimuli creation process.

To stimulate the four experimental conditions, four types of agents were created: agents in both low and high anthropomorphism levels, which were embodied virtually and physically. To manipulate anthropomorphism level, we followed the morphological way, which has been used in prior research (Goudey and Bonnin, 2016; Kang and Kim, 2020), and in current automobile manufacturers' practice (see Figure 1). Specifically, we created low-level anthropomorphized agents by including only one morphological characteristic of a head. High-level anthropomorphized agents were created through including more morphological characteristics, including a head, arms, legs, and a body. Moreover, as user responses are influenced by agents' gender, age, and personality (Cafaro et al., 2012; Lee et al., 2018; Ter Stal et al., 2020b), the involvements of these factors can possibly confound with the influences of anthropomorphism level and physicality. While creating agents, we attempted to design them in a neutral way to avoid any possible confounding influences. To do so, we firstly created line drawings of low-level and high-level anthropomorphized agents. Next, we created both virtual and physical agents (see Figure 3). For virtual conditions, the virtual agents were integrated into IVIS interfaces by arranging on the left-top part of the interfaces. For the physical conditions, the physical agents were positioned on the dashboard. The size of physical agents and virtual agents was kept as similar as possible.

**Figure 4:**
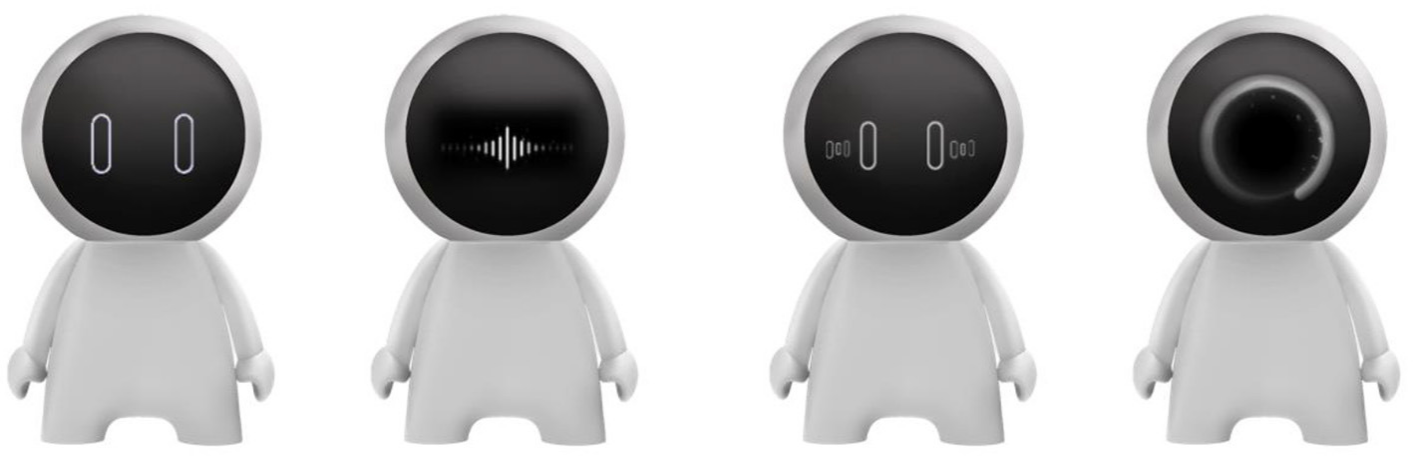
Four facial expressions exhibited by in-vehicle agents: waiting, listening, talking, and loading, in the condition of virtual high-level anthropomorphised agents.

To improve the realism of the created intelligent agents, we created different facial expressions according to system status, including waiting, listening, talking, and loading (see Figure 4). The agents support voice interaction with users, and the voice was created by a voice generator (www.xunjie.com) with a female voice version.

### Pre-test

The pre-test was conducted to examine the success of manipulations of anthropomorphism level. Specifically, a controlled experiment was conducted with anthropomorphism level as the between-subject factor and agents' physicality as the within-subject factor. Each participant was randomly assigned to one of the two conditions (low-level vs. high-level anthropomorphism). They were asked to evaluate two agents: virtual and physical agents. The order of presenting the virtual and physical agent was counterbalanced.

Forty participants were collected (mean age = 24.3, 62.5% male). Participants were firstly invited to the laboratory. Next, they were asked to view the agent (either virtual or physical agent) and then fill in questionnaires based on relatedness between stimuli and human. Specifically, as we manipulate anthropomorphism level by including a different number of morphological features, we measured anthropomorphism level by examining to what extent the agents facilitate users to relate to a person. They were asked to respond toward the following three-item statements from 1 (strongly disagree) to 7 (strongly agree): “after seeing this profile, I am confident to draw the conclusion that it resembles the image of a human,” “after seeing this profile, I am able to relate to the image of a human,” and “after seeing this profile, I can think of the image of a human immediately” (α = 0.916–0.964) (adapted from the measure of visual relatedness, adapted from Cheng et al., 2019).

One-way ANOVA was conducted with anthropomorphism level as independent variable and participants' ratings on visual relatedness in virtual and physical condition as dependent variables. Results showed that anthropomorphism level exerts significant influences on participants' ratings on visual relatedness across both virtual (*F* (1.38) = 19.69, *p* < 0.01) and physical conditions (*F* (1.38) = 15.92, *p* < 0.01). Specifically, in virtual condition, participants reported significant higher ratings of visual relatedness for the high-level anthropomorphised agent than the low-level one (M_high−level_ = 5.82 vs. M_low−level_ = 3.93). Similar results were found in physical condition (M_high−level_ = 5.18 vs. M_low−level_ = 3.35). Taken together, these results confirm the success of created stimuli.

### Procedure

The main experiment adopted a Wizard-of-Oz setup (Dahlbäck et al., 1993). Participants thought that the system automatically interacted with them, but the experimenter controlled the system in order to respond to participants simultaneously. While participants interacted with the system, the agent responded by conversational speech and different facial expressions. Meanwhile, IVIS interfaces presented the immediate information related to driving status. The voice and facial expressions were identical among four conditions. The only difference among the four conditions lay in the ways of embodied agents.

Upon arrival, we firstly welcomed participants and explained the aim and procedure of the experiment. Next, the driving simulator and IVIS with in-vehicle agents were introduced to them. They were also instructed on how to use the driving simulator and how to interact with the IVIS by voice interaction. They were given time for a trial on the simulator and an opportunity to interact with the agent until they confirmed that they acquired how to drive and interact with the agents. Next, before the main session, they were asked to view the agent carefully and respond to the questions related to visual relatedness that were identical to the measures in the pre-test. Their answers were further used for manipulation checks.

In the main session, all participants drove the car in the simulator along the set course. They were asked to drive steadily as they usually did in a real-life setting. During driving, participants were required to perform several tasks through interacting with agents by voice interaction. These interaction tasks include search for music, re-navigation, and send a message to a friend. The whole driving task was around 10 min. After completing the driving task, participants were asked to fill out a questionnaire to indicate their subjective perceptions of the IVIS and driving experience.

### Measures

We used questionnaires to measure participants' trust and their perceived control of their driving process. In order to improve reliability, multiple items were used to measure each construct. In the data analysis, the multiple items were combined by calculating the average score for each respondent. Following (Soh et al., 2009), the multidimensional trust scale was used in this study to measure participants' cognitive and affective trust in the IVIS integrated agent. Cognitive trust was measured by three 7-point Likert scales by rating the following statements “the system that I just used was credible/accurate/trustworthy” from 1 (strongly disagree) to 7 (strongly agree) (α = 0.937) (Lee et al., 2015; Kang and Kim, 2020). Affective trust was measured by asking participants to what extent they believe the following adjectives (i.e., positive, enjoyable, likable) can describe the IVIS anchored from 1 (describes very poorly) to 7 (describes very well) (α = 0.878) (Lee et al., 2015). Perceived control was measured by asking participants to indicate to what extent they agreed with the following two items: “when I interacted with the IVIS, I feel that usage procedure is completely up to me” and “when I interacted with the IVIS, I feel more control over it” on a 7-point Likert scale from 1 (strongly disagree) to 7 (strongly agree) (*r* = 0.731, *p* < 0.01) (adapted from (Wen et al., 2019; Anjum and Chai, 2020).

To validate the success of created stimuli, we included measures of visual relatedness, which are identical to those measures used in pre-test (α = 0.914). Moreover, prior research shows that users respond to attractive agents more positively (Khan and Sutcliffe, 2014). To control the possible confounding effects brought by agent attractiveness, we measured attractiveness by asking participants to indicate their opinions based on a 7-point scale on the following two items: “this agent looks unattractive/attractive” and “this agent looks ugly/beautiful” (*r* = 0.809, *p* < 0.01).

We also considered the possible influence of participants' familiarity of IVIS, which was measured by answering the following three questions: “to what extent are you familiar with knowledge on IVIS” from 1 (not at all familiar) to 7 (very familiar), “to what extent do you consider yourself have a good level of knowledge of IVIS” from 1 (no knowledge) to 7 (a lot of knowledge), and “to what extent do you consider yourself about IVIS” from 1 (very well-informed) to 7 (uninformed) (α = 0.845) [adapted from (Thompson et al., 2005)].

In addition to the subjective measures, the objective driving performance was assessed by the mean lane deviation data. Prior to start of each main session, the simulator was reset. The vehicle was centered on the lane without any deviation. During main sessions, any movements toward the center and edge line were recorded by the simulator automatically every second. Along the driving task, the average deviation is calculated, which is used to measure driving performance.

## Results

### Manipulation Check

To check the success of the manipulation of anthropomorphism level, one-way ANOVA was conducted with anthropomorphism level as independent variable and participants' ratings on visual relatedness as the dependent variable. Results showed that anthropomorphism level exerts a significantly positive influence on participants' rating on visual relatedness [*F* (1, 114) = 5.50, MSE = 2.015, *p* = 0.01, M _low−anthropomorphism_ = 4.25 vs. M _high−anthropomorphism =_ 4.87]. This result confirms that the manipulation of agents' anthropomorphism level is successful. To avoid confounding effects brought by attractiveness, two-way ANOVA was conducted with anthropomorphism level and physicality as independent variables, and participants' ratings on attractiveness as the dependent variable. No significant differences were detected in terms of the influences of anthropomorphism level (*p* > 0.10) or physicality (*p* > 0.10). These results ruled out the possibility of the confounding effect brought by attractiveness.

### The Influences of Anthropomorphism Level, Physicality, and Their Interaction (Responses to RQ 1,2,3)

Research questions 1–3 aimed to investigate the influences of anthropomorphism level and physicality and their interaction. Multivariate ANOVA was conducted with anthropomorphism level and physicality as independent variables and user perceived control, affective trust, cognitive trust, and driving performance as dependent variables. No significant influences of anthropomorphism level or physicality were detected for users' perceived control (*p* > 0.10), cognitive trust (*p* > 0.10), affective trust (*p* > 0.10), and driving performance (*p* > 0.10). However, the result revealed a significant interaction effect between anthropomorphism level and physicality on participants' ratings of affective trust [*F* (1, 112) = 5.43, MSE = 0.836, *p* = 0.022, η^2^ = 0.046], cognitive trust [*F* (1, 112) = 5.20, MSE = 0.649, *p* = 0.024, η^2^ = 0.044], and perceived control [*F* (1, 112) = 6.66, MSE = 1.10, *p* = 0.011, η^2^ = 0.056]. The interaction effect was not significant for driving performance [*F* (1, 112) = 0.268, MSE = 514.513, *p* = 0.61, η^2^ = 0.002].

**Figure 5:**
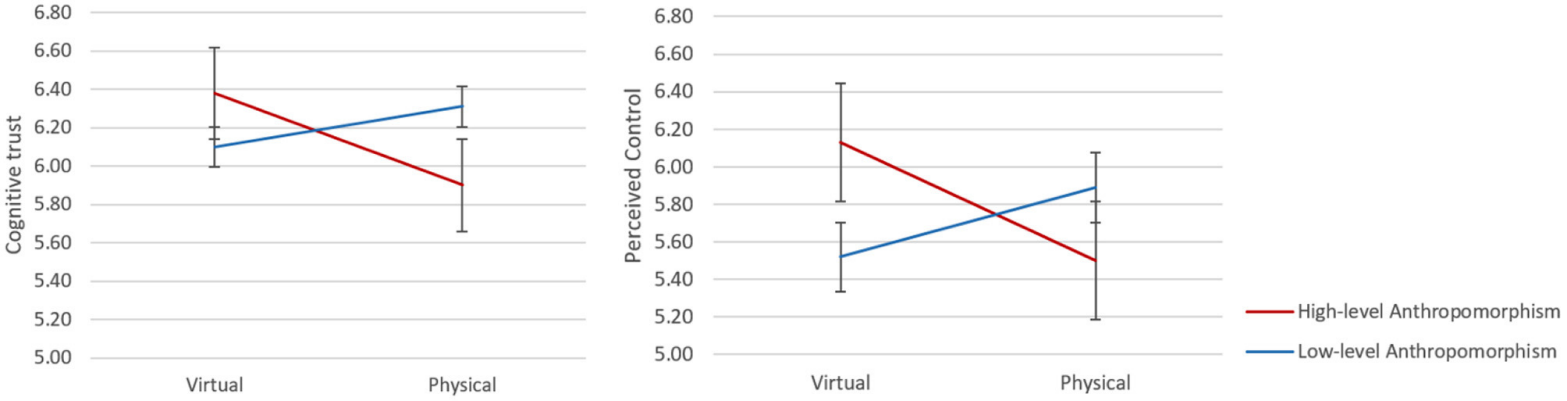
Interaction effects of anthropomorphism level and physicality of participants' cognitive trust and perceived control.

We conducted further analysis to interpret these interaction effects. Specifically, for agents with a high level of anthropomorphism, participants who interacted with virtual agents reported significantly higher ratings than the ones who interacted with physical agents in terms of cognitive trust [*F* (1, 58) = 5.77,MSE = 0.593, *p* = 0.020, η^2^ = 0.09] and perceived control [*F* (1, 58) = 6.85, MSE = 0.879, *p* = 0.011, η^2^ = 0.106] (see Table 1). In terms of affective trust, a marginal significant difference was found between virtual and physical condition [*F* (1, 58) = 3.59, MSE = 0.668, *p* = 0.063, η^2^ = 0.058]. But when interacting with low-level anthropomorphized agents, participants did not show significant differences between the virtual and physical conditions in terms of trust and perceived control (see Figure 5).

**Table 1:** Results of two-way ANOVA.

		**Affective trust**	**Cognitive trust**	**Perceived control**
		**Mean (SD)**	**Mean (SD)**	**Mean (SD)**
Low-level Anthropomorphism				
	Virtual	5.79 (1.04)	6.10 (0.93)	5.52 (1.24)
	Physical	6.19 (0.98)	6.31 (0.73)	5.89 (1.06)
High-level Anthropomorphism				
	Virtual	6.14 (0.74)	6.38 (0.64)	6.13 (0.84)
	Physical	5.74 (0.89)	5.90 (0.88)	5.50 (1.03)

### The Mediating Role of Perceived Control

**Figure 6:**
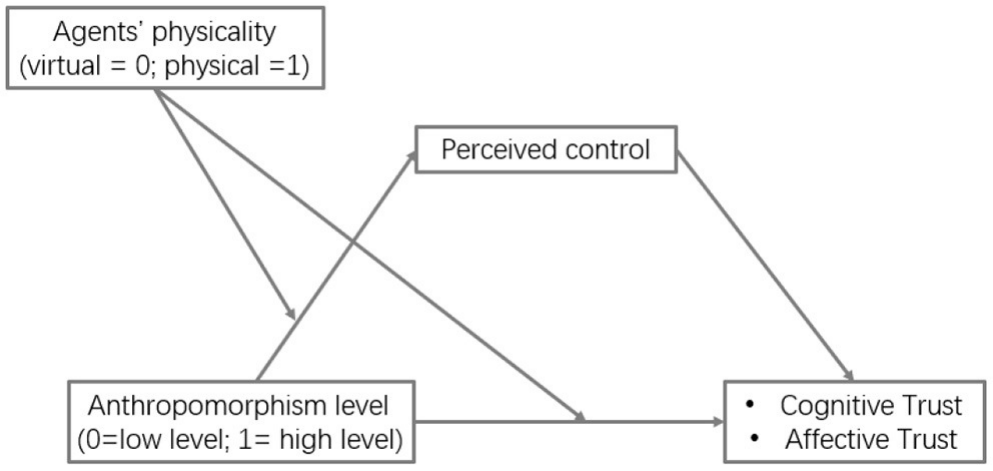
Moderated mediation test.

To further examine the mediating role of perceived control, we conducted a mediation analysis with MODMED model 8 (see Figure 6) by following the methodology proposed by Preacher and Hayes (2004). Results revealed that perceived control mediated the interaction effects of agents' anthropomorphism level and physicality on user cognitive trust (95% CI, −0.73– −0.08) and affective trust (95% CI, −0.92– −0.12) without including zero (Preacher and Hayes, 2004; Zhao et al., 2010). To examine the moderated mediation, we further explored the indirect effects for both anthropomorphism levels separately. For high-level anthropomorphism, the mediation through perceived control was significant for cognitive trust (B = −0.24, 95%CI, −0.44– −0.06) and affective trust (B = −0.31, 95%CI, −0.59– −0.08). Differently, for low-level anthropomorphism, the mediation through perceived control was not significant for cognitive trust (B = 0.14, 95%CI, −0.07–0.40) or affective trust (B = 0.18, 95%CI, −0.11–0.48) (see Table 2 for results). Taken together, these results demonstrated the mediator role of perceived control between the interaction effects of anthropomorphism level and physicality on users' cognitive and affective trust.

**Table 2:** Results of moderated mediation test.

**Dependent variable**	**Moderated mediation test Index (Boot SE), Boot 95% CI**	**Conditional indirect effect at values of anthropomorphism level** **B (Boot SE), Boot 95% CI**		**Direct effect B (Boot SE), Boot 95% CI**	
		**Low-level**	**High-level**	**Low-level**	**High-level**
Cognitive trust	Index = −0.38 (0.16)* CI = −0.73, −0.08	B = 0.14 (0.12) CI = −0.07, 0.40	B = −0.24 (0.10)* CI = −0.44, −0.06	B = 0.06 (0.19) CI = −0.31, 0.44	B = −0.24 (0.19) CI = −0.61, 0.13
Affective trust	Index = −0.49 (0.20)* CI = −0.92, −0.12	B = 0.18 (0.15) CI = −0.11, 0.48	B = −0.31 (0.13)* CI = −0.59, 0.08	B = 0.21 (0.20) CI = −0.20, 0.61	B = −0.09 (0.20) CI = −0.49, 0.31

*Statistically significant at 95% confidence level.

### The Moderating Role of Users' Familiarity With IVIS (Response to RQ4)

We conducted further analyses to learn the moderating role of users' familiarity. Participants vary in their familiarity with IVIS (M = 4.63; SD = 1.23). As users' familiarity is a continuous variable, the statistical power of dichotomizing can be low, which could possibly cause misleading interpretations (Irwin and McClelland, 2001; Fitzsimons, 2008). Thus, multiple regression analyses are strongly recommended to test the moderating effect rather than dichotomizing. This method has been used in examining the moderating role of individual characteristic in current literature (e.g., Mugge and Schoormans, 2012; Cheng and Mugge, 2022). There are three steps to conduct the analysis: (1) moderated regression analysis to learn the influences of IVs, moderators, and their interaction; (2) with significant interaction effects, spotlight analyses to learn to which group the influences of IVs are stronger. This analysis was conducted through replicating the regression analysis at low (-SD) and high level (+SD) of moderator value.

First, moderated multiple regression analyses were conducted to test the moderating effect of users' familiarity on the influence of anthropomorphism level and physicality. Specifically, we firstly created dummy variables of anthropomorphism level (0 = low-level anthropomorphism; 1 = high-level anthropomorphism), physicality (0 = virtual; 1 = physical), standardized users' familiarity, the interaction variable anthropomorphism level × physicality, the interaction variable anthropomorphism level × users' familiarity, the interaction variable physicality × users' familiarity. Next, these variables were used as independent variables. Finally, affective trust and cognitive trust were included as dependent variables. The regression analyses revealed a series of findings. As for cognitive trust, results revealed that it was significantly influenced by users' familiarity (ß = 0.485, *p* < 0.05), the interaction between anthropomorphism level and physicality (ß = −0.332, *p* < 0.05), and the interaction between anthropomorphism level and users' familiarity (ß = −0.249, *p* < 0.05) (see Table 3). These results replicate the interaction effects of anthropomorphism and physicality on cognitive trust. Results also showed that the influences of anthropomorphism level and physicality on cognitive trust significantly differ depending on users' familiarity with IVIS.

**Table 3:** Moderated regression with the dependent variables of affective and cognitive trust.

	**Cognitive trust**		**Affective trust**	
	**Standardized coefficients beta**	**Sig**.	**Standardized coefficients beta**	**Sig**.
Anthropomorphism	0.135	0.281	0.156	0.215
Physicality	0.086	0.504	0.169	0.192
Anthro * Physicality	−0.332	0.035**	−0.334	0.035**
Familiarity (z–score)	0.485	0.001**	0.461	0.001**
Familiarity * Anthro	−0.249	0.041**	−0.143	0.238
Familiarity * Physicality	−0.232	0.059*	−0.159	0.195

*p < 0.1; **p < 0.05.

**Figure 7:**
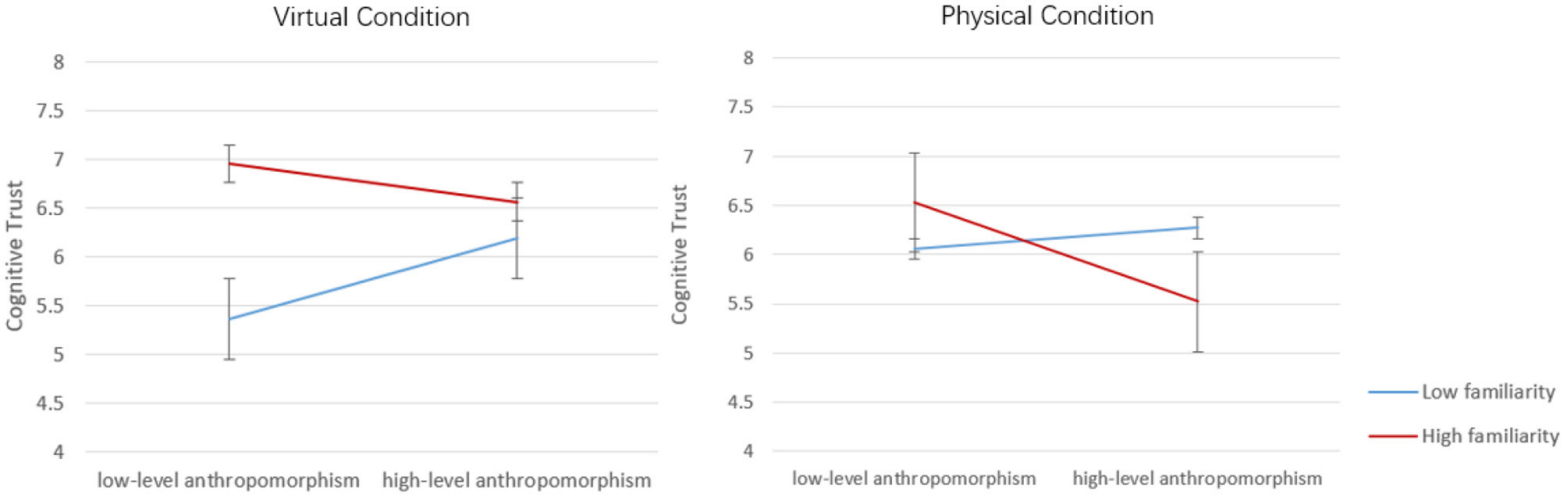
The moderating role of user familiarity on the influence of anthropomorphism level for virtual and physical conditions.

Next, to further interpret these interactions, we conducted spotlight analyses for users who are familiar with IVIS (one standard deviation above mean) and users who are unfamiliar with IVIS (one standard deviation below mean). For users who are not familiar with IVIS, anthropomorphism level makes a significant positive effect on user trust (b = 0.321, *p* < 0.05), but no significant effects were found on physicality (b = 0.258, *p*>0.10) (see Table 4). For users who are familiar with IVIS, no significant effects of anthropomorphism level (b = −0.052, *p*>0.10) or physicality (b = −0.086, *p*>0.10) were detected (see Table 4). These results were visualized in Figure 7. It can be clearly seen that anthropomorphism level makes a significant improvement for high familiarity users (blue line) in both virtual and physical conditions. But for users with high familiarity, the influence of anthropomorphism is not significant (red line).

**Table 4:** Spotlight analyses for users' familiarity with IVIS (+/-SD) on cognitive trust.

	**Users with low level of familiarity with IVIS**		**Users with high level of familiarity with IVIS**	
	**Standardized coefficients beta**	**Sig**.	**Standardized coefficients beta**	**Sig**.
Anthropomorphism	0.321	0.037**	−0.052	0.736
Physicality	0.258	0.104	−0.086	0.580
Anthro * Physicality	−0.332	0.035**	−0.332	0.035**
Familiarity (z-score)	0.485	0.001**	0.485	0.001**
Familiarity * Anthro	−0.312	0.041**	−0.310	0.041**
Familiarity * Physicality	−0.294	0.059	−0.284	0.059

*p < 0.1; **p < 0.05.

These findings suggest that the influences of anthropomorphism level on user cognitive trust differ from users' familiarity with IVIS. For users who are less familiar with IVIS, anthropomorphism level makes significant influences on cognitive trust. Differently, for users who are familiar with IVIS, anthropomorphism level and physicality make no significant influence on cognitive trust. Taken together, these results demonstrate the moderating role of user familiarity of IVIS.

## General Discussion

This research focused on the different ways of embodying agents integrated into IVIS in driving contexts, specifically the influences of agents' anthropomorphism level and physicality on users' perception and driving performances. Through a factorial experiment, results revealed that there was an interaction effect between anthropomorphism level and physicality on user cognitive trust, not on affective trust. It indicates that the two layers of agent embodiments (anthropomorphism level and physicality) promotes users' rational and systematic processing (Kim and Sundar, 2016), which further influence users' perception of information credibility given by IVIS. Furthermore, mediation analysis further shows that the interaction effect was mediated by perceived control. The influence of anthropomorphism level on user trust was significant for users who were unfamiliar with IVIS. For users who were familiar with IVIS, no significant influence of anthropomorphism was found. In addition, no significant differences were detected in terms of driving performance.

### Theoretical Contributions

Findings of this study have extended current literature in several ways. First, this study contributes to previous studies on exploring the influences of agent embodiment influences on user responses (Ziemke, 2001; Lee et al., 2006). How agent embodiments influence user responses has been explored in learning (Li et al., 2016) and e-commerce contexts (Luo et al., 2006). This study contributes to this line of studies by focusing on the context of driving. Moreover, extensive studies have explored the interaction effects of different agent embodiment layers on user responses in various contexts, such as the interaction of agency and anthropomorphism (Nowak and Biocca, 2004; Kang and Kim, 2020), the interplay of bodily appearance and movement (Castro-González et al., 2016), and the interaction of anthropomorphism and realism (Li et al., 2016). This study extends this line of studies by investigating the interaction influences of anthropomorphism level and physicality in driving contexts.

By manipulating the two factors, we have found interaction effects on user cognitive trust and the mediating role of perceived control, which suggest that users' relationship with systems is an important factor to consider while embodying agents. Specifically, in contexts where agents play a secondary role (e.g., assistant), a stronger sense of the agent being “another person” seems to be beneficial, such as in online shopping (Qiu and Benbasat, 2009) and learning contexts (Moundridou and Virvou, 2002; Chang et al., 2010). However, while users' agency becomes prominent, the stronger sense of “another person being” seems to make no significant or even negative influences, such as in decision making (Kulms and Kopp, 2019) and game-playing contexts (Kim et al., 2016). In driving contexts, the higher tendency to consider agents as social actors makes drivers no longer consider agents as driving assistants, but as “another person” who intends to take control of driving. As a consequence, users feel less perceived control and less trust in IVIS.

Furthermore, this study has demonstrated the mediating role of perceived control. The importance of perceived control in drivers' experience has been well-supported by current literature, which showed that some driving assisted functions, such as lane-keeping and speed controlling, reduce users' perceived control (Mulder et al., 2012; Navarro et al., 2016). Extending these studies, this research further shows that users' perceived control is not only influenced by objective functions but also agent embodiments. Consistent with the prior finding (Hoffmann and Krämer, 2013), users experience less control when they feel agents become more dominant. In addition to the presence of a smile (Kim et al., 2016) and the physical presence of a robot (Hoffmann and Krämer, 2013), a physically present high-level anthropomorphized agent can also create a dominated feeling, threatening drivers' sense of control.

In addition, this study reveals the moderating role of user familiarity on the effects of anthropomorphism level. Results showed that the positive influences of anthropomorphism are only significant for users who have less experience with IVIS. This finding is well-supported by the notion that the effects of anthropomorphism are moderated by users' characteristics (Epley et al., 2007; Złotowski et al., 2015). Prior research demonstrated the positive effects of anthropomorphism level on user trust, but these effects were found in the contexts of autonomous vehicles (Lee et al., 2015), where users have limited experience and knowledge. Instead, in driving contexts, in this study, users are equipped with rich knowledge and experience with IVIS because of the extensive training for driving licenses. Consequently, the influence of anthropomorphism on user trusts in IVIS is largely eliminated.

As for physicality, no significant effect on user trust was found. The effect of physicality on user cognitive trust only reaches marginal significance (*p* <0.10) when considering the moderating role of users' familiarity with IVIS. But in further spotlight analysis, no significant results were found. These findings contrast with the current findings related to physicality (Li, 2015; Mann et al., 2015). The size of physical agents might cause a possible explanation for this. In previous studies, the positive effects of physicality were found in the contexts of human-robot interaction (Lee et al., 2006; Mann et al., 2015) and the robots used in these studies are much larger. For instance, the robot used by Mann et al. (2015) was 450 mm tall and 320 mm wide, and Sony Aibo used by Jung and Lee (2004) was 293 mm tall and 180 mm wide. In related studies of driving contexts (Kraus et al., 2016; Lee et al., 2019), Social NAO was used, which was 273 mm tall and 275 mm wide. However, in this study, the low-level anthropomorphized agent was 59 mm tall and 58 mm wide, and the high-level anthropomorphized agent was 114 mm tall and 95 mm wide. Therefore, future research could replicate this study by increasing the agents' size, revealing a significant influence of physicality. Another possible reason is that the interaction tasks in this study did not require users to touch the physical agents. As suggested in previous studies (Jung and Lee, 2004; Hoffmann and Krämer, 2013), touch is required in users' interaction with agents to maximize the potential of physicality. Future research could explore the influences of agent physicality by involving touch required interactions or gestures interactions.

### Practical Implications

Results of this research offer important implications for practice. With the advancements of artificial intelligence in automobile interaction, an increasing number of agents have been developed to integrate into IVIS. It is an important decision for manufacturers in terms of how the agents should be embodied, for which the results of this study have provided recommendations. Designers should carefully consider how different ways of embodying in-vehicle agents may influence user trust. Designers can embody agents in high-level anthropomorphism or create physical embodiments, but designers should avoid embodying agents in high-level anthropomorphism and present agents physically, which can threaten drivers' sense of control while driving, leading to lower trust. Moreover, because of the neutral way of creating agents to avoid confounding effects, this study only detects influence of agent embodiments on cognitive trust not on affective trust. In practice, with alternative ways to create anthropomorphized agents, designers can possibly influence both cognitive and affective trust. As a result, users may feel the systems are credible and favorable.

Moreover, this study reveals the mediating role of perceived control. Besides anthropomorphism and physicality, many other factors may affect users' perceived control. For example, speech can take different styles, such as dry speech, formal command, informal conversion. The formal command may make drivers feel a lack of control. Therefore, designers and engineers should carefully assess the possible influences of these factors on users' sense of control.

The implications of the results of this study are not only limited to driving contexts but also applicable to agent embodiments in other contexts. One of the findings of this study is the importance of perceived control in users' driving experience. To what level users require perceived control depends on specific interaction contexts. As demonstrated by Kim and Mutlu (2014), users experienced robots differently when they had cooperation relationships than competition relationships. In smart home contexts, anthropomorphism largely alleviates users' feeling of losing control (Kang and Kim, 2020) because users demand a sense of connectedness in home contexts. Therefore, when embodying agents, developers and designers should firstly consider the relationships between users and agents in specific contexts, which determines to what extent users need the sense of control. Then, designers can explore optimal ways to embody agents to create the desired sense of control.

Another finding of this study lies in revealing the moderating role of users' familiarity with IVIS on the effects of anthropomorphism. This finding suggests that anthropomorphism strategy can be particularly effective for users' unfamiliar technologies, such as the voice interaction technology. Thus far, the adoption rate of voice interaction technology is around 40% (DBS interactive, 2022), indicating that most people may lack experience and knowledge of voice interaction. Especially for older adults, the adoption of voice interaction can help them reduce distraction in driving (Jonsson et al., 2005), but they show serious resistance (Heinz et al., 2013). In addition to improving users' trust, anthropomorphism strategy can bring other benefits, such as fostering social connectedness (Kang and Kim, 2020; Yang et al., 2020), which can even contribute to older adults' well-being.

### Limitations and Future Research

Although this research is carefully conducted, it carries some limitations, which might be improved in future research. First, while manipulating anthropomorphism levels, we followed the morphological method, in which the high-level anthropomorphized agents were created by including more morphological features. This manner allowed us to improve anthropomorphism levels while controlling the influences of other confounding effects. However, there can be alternative ways to manipulate anthropomorphism, such as improving realism (van Vugt et al., 2007) and including more anthropomorphic cues (e.g., intentions, empathy, emotions, etc.) (Lee et al., 2015). The involvement of these anthropomorphic cues can further improve anthropomorphism levels and make agents exhibit certain personalities (Hwang et al., 2013). Prior research suggested that people often respond to agents more positively when they exhibit similar personalities (Isbister and Nass, 2000; Braun et al., 2019). People are more likely to consider agents with similar personalities as teammates than competitors. Thus, it could be possible that the similarities between agents and users can eliminate users' feeling of lack of control found in this study, which could be interesting for future research.

Moreover, this study was conducted in a laboratory setting with a driving simulator. We measured both users' subjective perceptions with IVIS and objective driving performance by lane deviation. Although results did not reveal the significant influences of agent embodiments on objective driving performance, it does not mean agent embodiments make no influence on driving performance. In fact, the insignificant influence on driving performance might be caused by the simplified driving contexts and the short driving period in the experiment. Users can easily perform well in the simplified and short driving task. Differently, in real driving contexts, the traffic condition can be more complicated and drivers often drive for a longer time. Driving performance are more likely to be influenced by agents. More specifically, in addition to lane deviation, many alternative indicators are also important for driving safety, such as off-road glances, task completion time, and distractions. Future research can explore the influences in real driving contexts and examine whether agent embodiments can influence these indicators.

Furthermore, perceived control has received increasing research attention in the field of human-computer interactions. In this context, perceived control relates to one's perceived ability to affect the outcomes (Pacherie, 2007; Pacheco et al., 2013). This study reveals the mediating role of perceived control. It would be interesting for future research to manipulate perceived control directly, to further reveal its influence on users' performance and experience. In addition, to measure perceived control, we used two-item scales, which have been developed and used in previous studies (Anjum and Chai, 2020; Zafari and Koeszegi, 2020). However, these two items mainly capture users' own feelings over the systems while not considering the possible constraints made by systems, which could also influence users' perceived control. Therefore, it is worthwhile developing perceived control scales to capture users' subjective feeling from both aspects, which could be interesting for future research.

Another limitation lies in the sample. This study collected participants in a university. These participants are relatively young, and most participants majored in technology-related areas, such as computer science and electronic engineering. They are more likely to be familiar with IVIS and to accept in-vehicle agents. Moreover, although all the participants have driving licenses, they can still be different from people who drive every day for commute. Future research may replicate this study with the general population.

Furthermore, this study focused on a practical context of users driving the vehicle by themselves. However, self-driving vehicles are rising nowadays, which do not require drivers' interference during driving. It is particularly interesting to consider the role of users' sense of control in this context. In this case, drivers need to share controls with self-driving vehicles, and it is important for self-driving systems to assist drivers to fulfill their goals without hindering their sense of control (Wen et al., 2019). Probably, in self-driving status, agents need to exhibit prominence to convince drivers that the self-driving system is sufficiently powerful and reliable to drive safely. Differently, in the situations where users need to drive, agents may play an assistant role to let users feel complete control over driving. Future research could explore the dynamic role of agents, which could facilitate user trust, leading to the successful adoption of self-driving vehicles.

## Data Availability Statement

The raw data supporting the conclusions of this article will be made available by the authors, without undue reservation.

## Ethics Statement

Ethical review and approval was not required for the study on human participants in accordance with the local legislation and institutional requirements. The patients/participants provided their written informed consent to participate in this study.

## Author Contributions

PC and JY: conceptualization and hypotheses development. FM and YW: stimuli creation. FM: data collection. PC: data analysis and draft preparation. JY: revision and editing. All authors contributed to the article and approved the submitted version.

## Funding

This work was supported by the National Natural Science Foundation of China (Grant No. 72002057), Humanities and Social Science Projects of the Ministry of Education in China (Grant No. 20YJC760009), National Social Science Fund of China (Grant No. 16BSH097), and Shenzhen Basic Research Program (Grant No. JCYJ20190806142401703).

## Conflict of Interest

YW was employed by the company Kuaishou Technology. The remaining authors declare that the research was conducted in the absence of any commercial or financial relationships that could be construed as a potential conflict of interest.

## Publisher's Note

All claims expressed in this article are solely those of the authors and do not necessarily represent those of their affiliated organizations, or those of the publisher, the editors and the reviewers. Any product that may be evaluated in this article, or claim that may be made by its manufacturer, is not guaranteed or endorsed by the publisher.
